# Hatching Egg Sanitizers Based on Essential Oils: Microbiological Parameters, Hatchability, and Poultry Health

**DOI:** 10.3390/antibiotics13111066

**Published:** 2024-11-09

**Authors:** Gabriel da Silva Oliveira, Concepta McManus, Pedro Henrique Gomes de Sá Santos, Davi Emanuel Ribeiro de Sousa, José Luiz de Paula Rôlo Jivago, Márcio Botelho de Castro, Vinícius Machado dos Santos

**Affiliations:** 1 Faculty of Agronomy and Veterinary Medicine, University of Brasília, Brasília 70910-900, Brazil; 2 Center for Nuclear Energy in Agriculture (CENA), University of São Paulo, São Paulo 13416-000, Brazil; 3 Institute of Biological Sciences, University of Brasília, Brasília 70910-900, Brazil; 4 Federal Institute of Brasília—Campus Planaltina, Brasília 73380-900, Brazil

**Keywords:** *Allium sativum*, *Citrus aurantifolia*, egg incubation, eggshell, egg microbiology, essential oils, hatching eggs, natural products, *Ocimum basilicum*, scanning microscopy

## Abstract

**Background:** Eggshell contamination threatens the viability of hatching eggs. This contamination can be caused by harmless, opportunistic, or pathogenic bacteria. Although necessary, the use of synthetic antibiotics to treat eggshells can present several significant problems: They can be toxic and damage the shell, and, most worryingly, they can lead to bacterial resistance. Faced with these challenges, the objective of this research was to create and test a sanitizing plan for hatching eggs using essential oils derived from *Citrus aurantifolia* (CAEO), *Ocimum basilicum* (OBEO), or *Allium sativum* (ASEO). **Methods:** Sanitizing solutions containing specific concentrations of these essential oils were prepared, and their antimicrobial properties and contributions to poultry safety and hatching parameters were investigated. **Results:** The bacterial load was reduced in eggshells sanitized with essential oils, and the degree of bacterial inhibition, along with their safety profile, may be directly related to optimal hatchability rates, lower incidences of contaminated dead embryos, and the hatching of healthy chicks. **Conclusions:** Together, these results reinforce the importance of essential oils in the development of effective and safe treatments for managing hatching eggs.

## 1. Introduction

**Figure 1:**
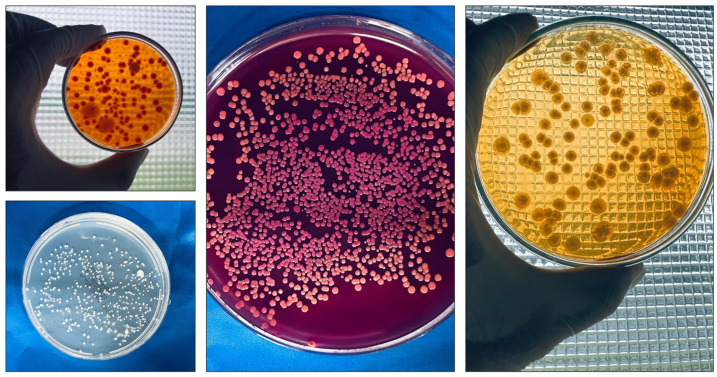
Bacterial colonies grown on different culture media from samples collected from eggshells (immediately after collection).

Embryonic development is a complex physiological process characterized by intricate communication between the internal and external structures of eggs. This sophisticated dialog between structures is essential for the proper development of the embryo. However, the interactions between the eggshell and the embryo present a significant duality. Although these interactions can promote embryonic development, they can also be harmful. The eggshell, as it is an interface with the external environment, is subject to the presence of various contaminants, including pathogenic microorganisms [1,2]. Microbiological tests confirmed the presence of microbial contamination on the eggshell post collection (Figure 1). This contamination can compromise the integrity of the embryo, negatively impacting its development [3]. Sanitary practices are needed to mitigate the risk of bacterial contamination in hatcheries, feed and input storage areas, and facilities supporting poultry activities, such as administrative areas, bathrooms, changing rooms, laboratories, and warehouses. The same attention should be given to production facilities, sanitization rooms, egg storage areas, egg transport vehicles, and personal hygiene practices, with particular emphasis on sanitizing hatching eggs.

Sanitizers used in poultry farming are normally formulated with a synthetic chemical base (e.g., formaldehyde, glutaraldehyde, or quaternary ammonium) and have been proven to be efficient at reducing the level of contamination by pathogenic bacteria that cause fatal infections [4,5,6,7]. On the other hand, these sanitizers can also be associated with health problems ranging from allergic skin reactions to the development of malignant cancer [8,9,10,11]. This problem worsens when companies do not warn users of their sanitizers that they must handle these with personal protective equipment. Poultry researchers, teachers, and professionals play vital roles in the theoretical and practical education of poultry producers and managers about advanced sanitization techniques, adherence to healthy sanitizers, modification of harmful protocols, and immediate recognition of complications and irregularities in practice.

The selection of hatching egg sanitizers that contain compounds derived from plants, such as essential oils, can be a beneficial decision, considering the results of reviews, published in renowned scientific journals, on the benefits of essential oils for the sanitization of hatching eggs and the protection of human health [12,13,14,15,16,17,18]. Oliveira et al. [12] suggested the use of essential oils as sanitizing agents for hatching eggs, emphasizing their safety and effectiveness in reducing the microbial load on eggshells, which can significantly increase hatchability rates. Lesgards et al. [18] favored the advancement of essential oils in medical research, highlighting that these compounds may exhibit advanced anticancer properties by inducing cell death in cancer cells without affecting normal cells.

**Figure 2:**
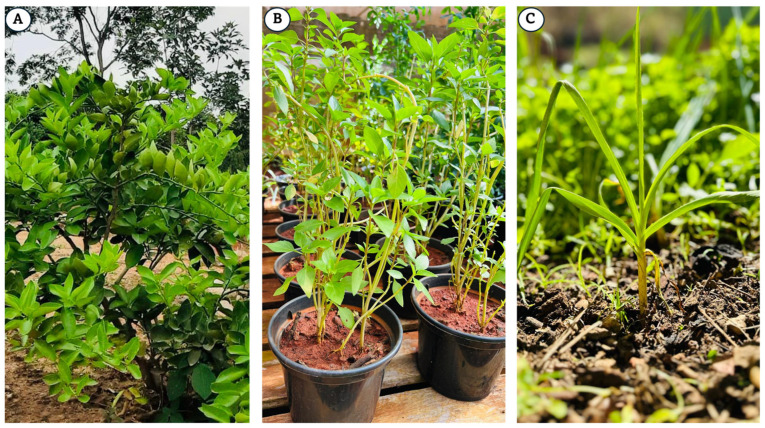
*Citrus aurantifolia* (**A**), *Ocimum basilicum* (**B**), and *Allium sativum* (**C**) plants.

The plants *Citrus aurantifolia* (Tahiti lemon), *Ocimum basilicum* (basil), and *Allium sativum* (garlic) (Figure 2) are among the best-known sources of essential oils worldwide. *Citrus aurantifolia* is a plant that can reach an average height of 5.10 m and a canopy area of approximately 17.50 m^2^, with an estimated volume of 105.16 m^3^. The leaf area can reach 16.53 cm^2^. Each tree can produce an average of 405 fruits, with a fruit set rate of 30.95%, yielding approximately 18.64 kg/tree. The number of flowers/clusters can reach 7.00, and the number of fruits/clusters can reach 2.17. The fruits can have a juice content of approximately 39.89%, with total soluble solids measuring 7.64 °Brix. The acidity can reach 7.94%. Each fruit may contain an average of 24.49 mg of ascorbic acid/100 mL of juice. The polar diameter of the fruits is 3.31 cm, the equatorial diameter is 3.17 cm, and the average volume is 42.36 mL. The average peel thickness can reach 2.33 mm [19]. *Ocimum basilicum* is a plant that can reach an average height of 53.4 cm, with approximately 16.0 branches/plant. The plant diameter can reach approximately 41.0 cm. The leaf blade has an average length of 6.4 cm and a width of 3.4 cm. The time to flowering can reach 72 days, with approximately 32.2 inflorescences per plant, each averaging 15.6 cm in length, and approximately 11.1 clusters/main stem. In terms of the yield, the average plant weight can reach 153.4 g. The yield of fresh herbs can reach 16.6 kg/100 m^2^, whereas the yield of air-dried herbs can reach 3.5 kg/100 m^2^. The plant can also contain an essential oil content of 1.03% and has an essential oil efficiency of 0.31 g per plant [20]. *Allium sativum* can reach an average height of 67.40 cm and produces approximately 7.80 leaves per plant, with an average leaf length of 34.20 cm and a leaf width of 3.01 cm. The bulb can have a neck thickness of 1.45 cm and a diameter of 3.70 cm. The bulb yield per plant can average 25.33 g, with approximately 20.55 cloves per bulb. Each clove weighed an average of 2.62 g, with a length of 1.01 cm and a diameter of 1.04 cm. The total soluble solids can reach approximately 36.01% [21].

The essential oil extracted from the *Citrus aurantifolia* (CAEO) plant is an aromatic natural compound that is predominantly composed of hydrocarbons in the form of monoterpenes and sesquiterpenes, with D-limonene as the main component [22]. This essential oil was effective in inhibiting in vitro bacterial strains, such as *Escherichia coli*, *Pseudomonas aeruginosa*, *Staphylococcus aureus*, and *Bacillus subtilis*, in addition to the fungus *Candida albicans* [22]. The essential oil extracted from *Ocimum basilicum* (OBEO) is a light yellowish and volatile substance, which composition is dominated by monoterpenes, sesquiterpenes, and phenylpropanoids, with estragole as the main constituent [23]. Exhibiting potent antimicrobial activity in vitro, this oil is effective against methicillin-resistant *Staphylococcus aureus*, *Staphylococcus aureus*, *Staphylococcus epidermidis*, *Enterococcus faecalis*, *Bacillus subtilis*, *Bacillus cereus*, *Escherichia coli*, *Pseudomonas aeruginosa*, *Acinetobacter baumannii*, *Salmonella* spp., *Klebsiella pneumoniae*, and *Candida albicans* [23]. The essential oil extracted from *Allium sativum* (ASEO) is a bioapplicable natural chemical solution that stores several bioactive compounds. This essential oil has demonstrated significant in vitro antibacterial activities against several bacterial strains, including *Escherichia coli*, *Pseudomonas aeruginosa*, *Staphylococcus aureus*, and *Listeria innocua* [24]. Remarkable findings were obtained from in vivo tests, where CAEO, OBEO, and ASEO showed robust abilities to substantially decrease the bacterial loads on the shells of hatching eggs [14]. This reduction was particularly evident with mesophilic and enterobacteria [14].

The antimicrobial properties of CAEO, OBEO, and ASEO are not inferior to those of commercial sanitizers [14]. This not only promotes a substantial reduction in microbial contamination but also can encourage the production of next-generation antimicrobials based on CAEO, OBEO, and ASEO, with the capacity to promote green poultry farming [25]. As we continue to explore and better understand these essential oils within the poultry industry, new findings have emerged to strengthen the development of advanced natural antimicrobial solutions capable of meeting the needs of the poultry industry, which is increasingly aware of the importance of sustainability. Therefore, this study aimed to provide a thorough evaluation of the effects of CAEO, OBEO, and ASEO on hatching eggs. This assessment ranges from evaluating the microbiology of eggshells after the application of these oils to analyzing newly hatched chicks. This study seeks to fill gaps in the understanding of the potential benefits of these essential oils in the face of the microbial challenges faced by poultry farms in the management of hatching eggs.

## 2. Results and Discussion

The main options for controlling the bacterial contamination of eggshells on commercial poultry farms are based on conventional therapies, such as fumigation with formaldehyde (FA) [6]. However, this sanitization model can lead to adverse effects, as both poultry and humans cannot tolerate constant exposure, especially at high concentrations, because of potential health risks [26,27,28,29]. Sanitizing eggshells is essential for bacterial control, but the sanitizers used in this process raise significant concerns regarding health and safety. In our previous study [14], we reported that CAEO, OBEO, and ASEO effectively reduce the bacterial loads on eggshells. In this study, we expand this investigation and demonstrate how approaches based on CAEO, OBEO, and ASEO can influence the hatching and health parameters of poultry.

We began our study with a series of analyses to characterize the hatching eggs. This step was crucial to ensure that the eggs were suitable for subsequent evaluations and to minimize potential biases that could affect the study’s primary objective. The qualities and microbiological characteristics of the eggs are detailed in Table 1. After confirming that the eggs met the expected normal parameters, we proceeded with the subsequent stages of the study.

To elucidate the antibacterial effects of the essential oils on the eggshells, we counted mesophilic bacteria and Enterobacteriaceae at three distinct time points: once before incubation and twice during the incubation process. Overall, we found that compared with the other treatments, the essential oils were notably more effective (*p* < 0.05) at reducing eggshell contamination (Table 2). In addition to significantly lowering the initial bacterial counts, the essential oils demonstrated superior control of contamination recurrence, keeping the bacterial loads stable until the 18th day of incubation. The contamination levels observed on eggshells sanitized with the essential oils during incubation were lower than that in the reference treatment (FA), with the essential oils showing significantly reduced loads of mesophilic bacteria and Enterobacteriaceae on the 18th day of incubation. Until the ninth day of incubation, the eggshells treated with CAEO and ASEO were free of Enterobacteriaceae. On the ninth day, the Enterobacteriaceae loads were minimal on eggs sanitized with OBEO but did not significantly differ from those in the groups treated with the other essential oils. On the other hand, eggshells sanitized with FA, the standard model from the supplier farm, were less effective at preventing recontamination, as they presented significant increases in mesophilic bacteria and Enterobacteriaceae loads during incubation. The ASEO was the most effective at reducing the bacterial contamination of the eggshells.

More specifically, the essential oils reduced, on average, 70.53% of the mesophilic bacterial loads, and no Enterobacteriaceae were detected during the pre-incubation period. On the ninth day, the average reductions were 66.21% for mesophiles and 92.97% for Enterobacteriaceae. On the 18th day, the reductions were 62.89% for mesophiles and 73.54% for Enterobacteriaceae. These data are consistent with the findings presented in our previous review [12], where we demonstrated that on the basis of various studies, essential oils can reduce the number of mesophilic bacteria by up to 80.77%. These results are also supported by previous analyses of hatching egg sanitizers based on *Lavandula angustifolia* [30], *Origanum vulgare* [31], and *Cuminum cyminum* [32]. The reasons why essential oils kill bacteria are well documented. The main reasons for this include the inhibition of biofilm formation; the induction of apoptosis mediated by oxidative stress; the disruption of DNA synthesis; the leakage of intracellular proteins, ATP, and nucleic acids; and changes in the metabolic profile, which encompasses amino acid restriction and disturbances in energy metabolism [33,34].

**Figure 3:**
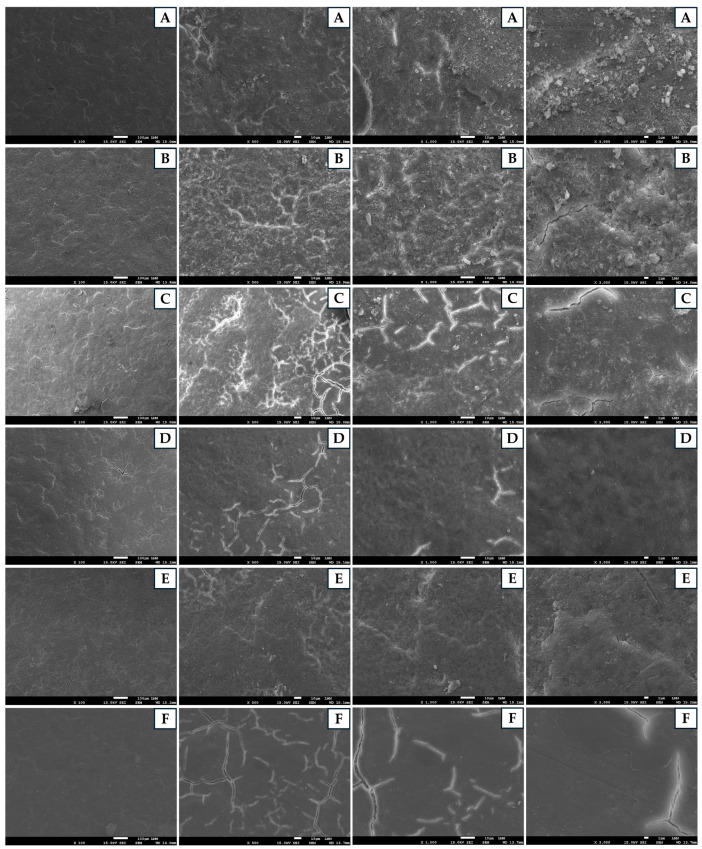
Scanning electron microscopy photographs of eggshells after different sanitization processes. ((**A**) non-sanitized eggs—NE): high degree of integrity and minimal degradation. ((**B**) grain alcohol—GA): low degree of integrity, severe degradation, and evident structural failure. ((**C**) formaldehyde—FA): critical degree of integrity, severe degradation, and risk of structural failure. ((**D**) *Citrus aurantifolia* essential oil—CAEO): reduced degree of integrity, significant cracks, and irregular texture. ((**E**) *Ocimum basilicum* essential oil—OBEO): moderate degree of integrity and some cracks and particles. ((**F**) *Allium sativum* essential oil—ASEO): highly reduced degree of integrity, many cracks, and many particles.

On the basis of the ultrastructural analysis of the eggshells after sanitization, we observed that non-sanitized eggshells maintained remarkably preserved structural integrity, with few visible flaws and a consistently smooth surface texture (Figure 3). The cracks observed on the eggshell surface were minimal, small in extent, and scattered, with no significant interconnectivity. The surface appeared uniform, with a smooth texture and few irregularities or aggregates. There were almost no visible particles or contaminants, suggesting a clean and intact surface with minimal signs of wear. On the other hand, eggshells sanitized with essential oils exhibited cracks ranging from few and superficial to numerous and deep, with from minimal to significant interconnectivity. The surface homogeneity also varied, ranging from good uniformity to a marked lack of uniformity. The presence of particles on the eggshell surface varied from a few to a substantial number. The wear on the shells ranged from mild to severe. Specifically, eggshells sanitized with OBEO showed no signs of advanced degradation, whereas those treated with CAEO and ASEO presented considerable stress and surface deterioration. We suggest that a significant portion of the effects observed on eggs sanitized with essential oils was associated with the GA used as a solvent. Eggshells sanitized solely with GA presented deep, interconnected cracks covering a large part of the surface, characterized by poor homogeneity, a highly irregular texture, and a marked presence of defects. The significant number of particles indicated degradation and contamination, with clear signs of structural failure on the surface. Eggshells sanitized with FA showed significant patterns of cracks that increased in complexity as the magnification of the analysis increased. The cracks were deep, interconnected, and present throughout the surface. This surface was the least homogeneous among all the treated surfaces, with an extremely irregular texture and signs of failure in multiple areas. The presence of particles was widespread and intense, indicating a high likelihood of contamination and/or internal degradation. The wear of the eggshells in this treatment group was extreme, with severe degradation, and the structure appeared to be on the brink of complete failure. Therefore, as expected, the non-sanitized eggshells presented the best scenario in terms of the integrity, whereas the eggshells sanitized with FA were the most compromised, severely affecting the structural integrity.

Overall, the incubation process was concluded at 516 h and 12 min, but the first hatch occurred at 469 h and 49 min, whereas the last hatch occurred at 501 h and 27 min, covering a period of 31 h and 38 min. This hatch window is within the expected range for commercial setters [35]. Previously, an incubation cycle involving non-sanitized eggs and those sanitized with ethyl alcohol, essential oils, or FA lasted an average of 31 h and 45 min [36].

We quantified the eggs’ weight loss based on the eggs’ initial weights and observed that the eggs sanitized with the essential oils did not show weight losses different from those of the non-sanitized eggs (Table 3). In contrast, the eggs sanitized with FA showed weight losses that exceeded the ideal limit required to ensure an adequate air cell by the end of embryonic development [37]. Losses of this magnitude have a significant impact on embryonic development; for example, excessive internal water loss can cause severe dehydration in the embryo [37]. The elevated weight losses of the eggs sanitized with FA corroborate the observation that it was the sanitizing agent that most compromised the integrity of the eggshell (Figure 3). In contrast, the damage caused by the essential oils to the eggshells did not result in weight losses outside the normal range observed for eggs treated with these oils (Figure 3). One possible explanation for this phenomenon is that despite the combination of alcohol and oil causing damage to the shells, the oily and residual characteristics of the essential oils, even at low concentrations, may have minimized the significant weight loss. Importantly, in recent years, essential oils have been used in egg coatings to improve their structures and reduce weight loss in eggs stored for more than 20 days [38,39,40].

To gain insights into hatching outcomes, we analyzed the hatchability rate of fertile eggs in response to the sanitizers applied to the eggshells (Table 3). First, we observed that under the same incubation conditions, the conventional FA-based sanitizer significantly reduced (*p* < 0.05) the hatchability rate, whereas the essential oils promoted the highest hatchability rates. Our hatchability results are in close alignment with previous findings on the sanitization of eggs using essential oils of *Syzygium aromaticum* at a concentration of 0.39% [41] and *Cuminum cyminum* and *Origanum vulgare*, each at 0.5% [32]. Compared with non-sanitized eggs, the application of these essential oils led to significant increases in hatch rates: for every 320 incubated eggs, 34 additional eggs successfully hatched. Similarly, for *C. cyminum* and *O. vulgare*, for every 50 incubated eggs, 6 more eggs hatched than in the untreated control group. Oliveira et al. [13] conducted a review that contrasted the effects on hatchability of eggs sanitized with FA and essential oils. The review revealed that on average, essential oils do not produce inferior results compared with FA in terms of hatchability.

**Figure 4:**
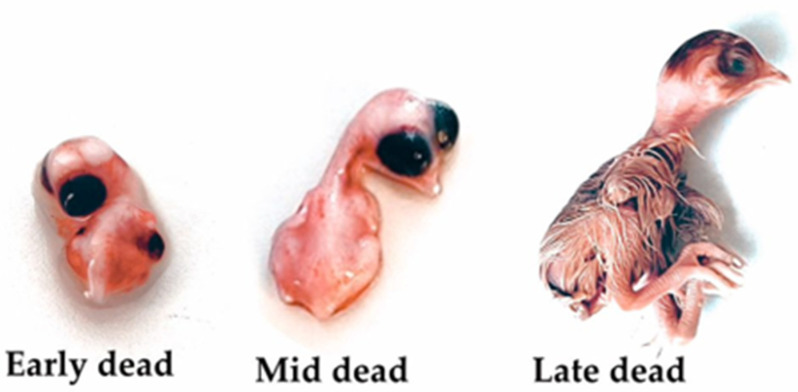
Dead chick embryos at different stages of development. The stages are categorized as follows: (Early) 0–7 days of incubation, (Mid) 8–18 days of incubation, and (Late) 19–21 days of incubation.

Embryonic mortality can be divided into three phases (Figure 4). We compared the mortality rates among the treatments. FA increased (*p* < 0.05) the rate of early embryonic mortality compared with both non-sanitized eggs and those treated with GA and essential oils (Table 3). Considering the average rate of early mortality associated with essential oils, we demonstrated that compared with FA, these oils reduced the mortality rate during this period by approximately 75%. The high early mortality rate observed in eggs sanitized with FA can possibly be attributed to the residual presence of this compound on the eggshell during the first days of development or because it can fix itself to the internal contents of the eggs. Similarly, Bekhet [42] reported an increase in the early embryonic mortality rate for eggs sanitized with FA. No differences were found for intermediate or late mortality rates among the treatments. The highest number of contaminated embryos was expected for the non-sanitized eggs, as these eggs are exposed to a more significant bacterial challenge than are the sanitized eggs.

To the best of our knowledge, our study is pioneering in linking the sanitization of hatching eggs with essential oils and the level of bacterial contamination in the yolk sac. Our findings indicate that interventions with CAEO, OBEO, and ASEO reduced (*p* < 0.05) the mesophilic bacterial load by 55.36% and the Enterobacteriaceae load by 56.98% in the yolk sacs of 18-day-old embryos (Table 4). Our hypothesis is that the use of essential oils helps to maintain low bacterial levels on the eggshell, thereby impeding the penetration of contaminating bacteria into the embryo. In this way, the embryo benefits from the antibacterial barrier created by the essential oils on the eggshell. Li et al. [43] corroborated our findings. They did not isolate nalidixic-acid-resistant *E. coli* in any of the yolk sac samples from chicks hatched from eggs artificially contaminated with bacteria on the shell and subsequently sanitized with 1.5% lysozyme. In contrast, when sanitization was performed with distilled water instead of lysozyme, 40% of the yolk sac samples contained *E. coli*. Although partial, our recent findings also provide important support for our previous hypothesis that the sanitization of hatching eggs with essential oils contributes to significant reductions in the microbial loads of newly hatched chicks [44]. We also noticed that a significant number of dead embryos due to contamination were identified in the non-sanitized eggs, whereas no mortality was recorded among embryos from the eggs sanitized with OBEO or ASEO (Table 3). Thus, we demonstrated the relationships among the practice of sanitizing eggshells, the reduction in bacterial counts on the eggshells, and the survival rates and bacterial qualities of the embryos. FA did not have the potential to reduce the bacterial loads in the yolk sacs of the embryos.

**Figure 5:**
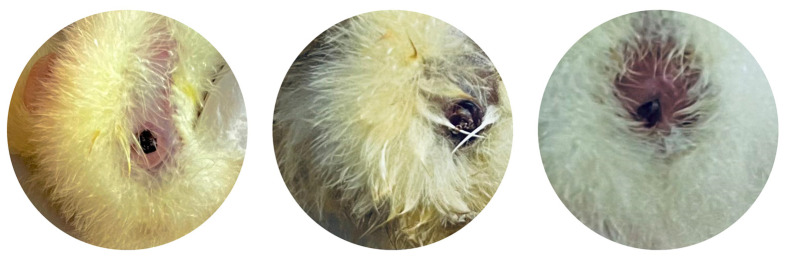
Navels of newly hatched chicks with poor healing.

Although the essential oils did not influence the chick weights, FA significantly reduced (*p* < 0.05) the chick weight compared with those of chicks from the non-sanitized eggs. This reduction is probably because of the degree of embryo dehydration caused by excessive egg weight loss during incubation because of eggshell damage. According to Aviagen [45], the low weights of chicks compared with what is expected on the basis of the weight of the eggs, indicating poor yield, is associated with chick dehydration, reduced yolk reserves, and excessive levels of activity and noise. Compared with the non-sanitized eggs, the hatching eggs sanitized with the essential oils tended to produce healthier chicks, with a significant difference observed only for OBEO (Table 5). To identify the main cause of the decline in chick quality from the non-sanitized eggs, we conducted a detailed analysis of individual factors (reflex, navel, legs, and beak). The analysis revealed that poor navel healing was the primary factor responsible for the reduced quality of the chicks hatched from the non-sanitized eggs (Figure 5). These findings highlight the importance for sanitizing hatching eggs. Among the treatments, OBEO produced the healthiest chicks.

**Figure 6:**
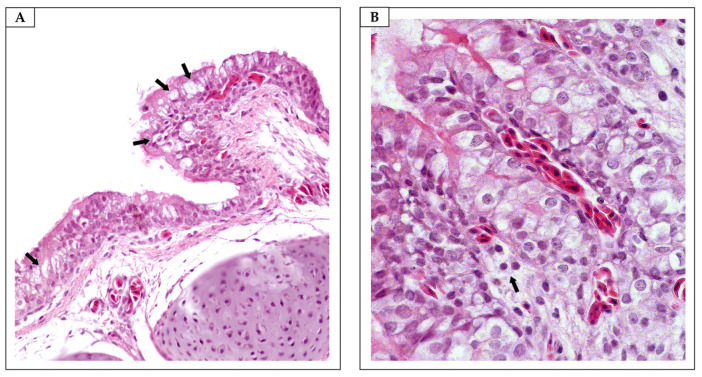
Tracheae. (**A**) Formaldehyde (FA) group. Multifocal goblet cell hyperplasia (arrows) (H&E, objective 40×). (**B**) FA group. Mononuclear inflammatory infiltration (arrows) (H&E, objective 40×).

**Figure 7:**
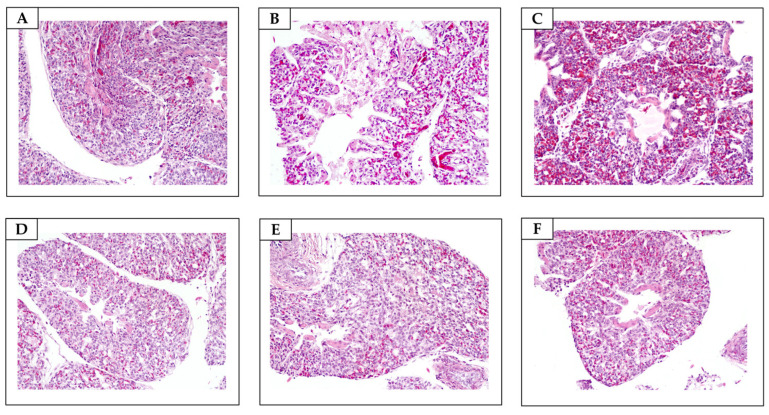
Lungs. (**A**) Non-sanitized eggs (NE). Unremarkable microscopic changes (H&E, objective 20×). (**B**) Grain alcohol (GA). Unremarkable microscopic changes (H&E, objective 20×). (**C**) Formaldehyde (FA). Severe pulmonary congestion (H&E, objective 20×). (**D**) *Citrus aurantifolia* essential oil (CAEO). (**E**) *Ocimum basilicum* essential oil (OBEO). (**F**) *Allium sativum* essential oil (ASEO). Unremarkable microscopic changes (H&E, objective 20×).

FA caused morphological changes, ranging from mild to moderate, in the tracheae and lungs of the embryos in all the embryo samples (Table 6; Figure 6 and Figure 7). Our findings corroborate the results of histological analyses carried out by Turkish researchers, who, approximately 16 years ago, confirmed that sanitizing hatching eggs with FA has adverse effects on the embryos’ tracheae [46]. Given the ability of FA to penetrate eggshells after exposure [27], especially owing to the damage caused to the shells’ integrity, we suggest that there may be an accumulation of toxic chemical compounds within the eggs’ internal compartments, with implications that extend throughout embryonic development. Although we previously suggested that proper sanitization of hatching eggs with FA could prevent adverse effects on embryonic development [44], our recent results highlight the need for further investigation to understand the factors that favor the toxic effects of FA on embryos, even when applied under appropriate conditions. To advance this field, we propose a multifactorial study on the sanitization of hatching eggs with FA that considers the characteristics of eggshells from different genetic strains; the purity, formulation, and quality of the FA; and the genetic variation of the embryos.

**Figure 8:**
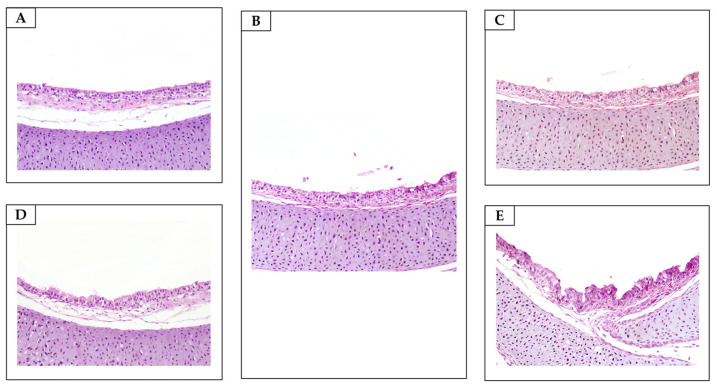
Tracheae. Unremarkable microscopic changes (H&E, objective 20×). (**A**) Non-sanitized eggs (NE). (**B**) Grain alcohol (GA). (**C**) *Citrus aurantifolia* essential oil (CAEO). (**D**) *Ocimum basilicum* essential oil (OBEO). (**E**) *Allium sativum* essential oil (ASEO).

Unlike those treated with FA, trachea and lung samples from embryos in the essential oil group presented no detectable morphological changes (Table 6, Figure 7 and Figure 8). These findings suggest that the potential penetration and accumulation of essential oils in the embryonic environment do not reach levels capable of inducing morphological alterations in the poultry respiratory system. Oliveira et al. [13] also demonstrated that chicks hatched from eggs sanitized with *Syzygium aromaticum* essential oil did not exhibit histopathological changes in the trachea. This allows us to speculate that although poultry have tissues with varying degrees of sensitivity, the embryonic mortalities observed in the group exposed to the essential oils were not attributable to toxicity associated with the egg sanitization process using these natural compounds. This conclusion is supported by previous studies that reported the absence of histopathological alterations in the digestive, cardiovascular, and central nervous systems and muscular tissues of one-day-old chicks hatched from eggs sanitized with essential oils [13,32].

**Figure 9:**
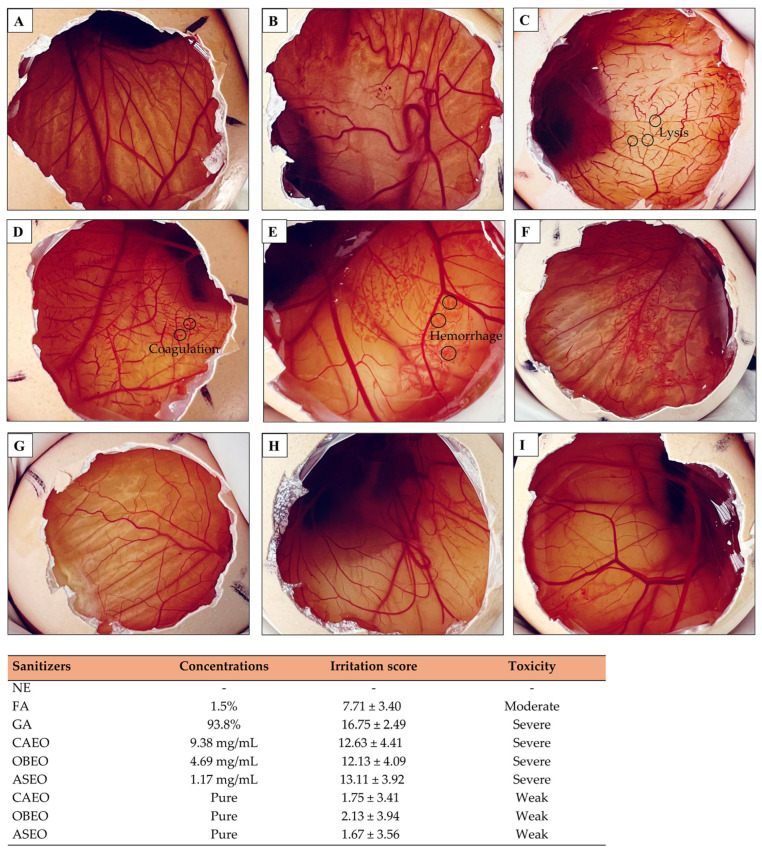
Photographic sequences of the hen’s egg test chorioallantoic membrane (HET-CAM) assay illustrating the CAM without the application of any sanitizing product (NE) (**A**) and the CAMs after contact with different sanitizers: formaldehyde (FA) (**B**), grain alcohol (GA) (**C**), *Citrus aurantifolia* essential oil (CAEO) (**D**), *Ocimum basilicum* essential oil (OBEO) (**E**), and *Allium sativum* essential oil (ASEO) (**F**). Additionally, the figure shows the membranes exposed to pure CAEO (**G**), OBEO (**H**), and ASEO (**I**). The irritation score is presented as the mean ± standard deviation of eight eggs, highlighting the toxic/irritant effects of each tested substance.

Changes in the chorioallantoic membrane (CAM) became visible after the application of the sanitizing solutions (Figure 9). GA caused lysis in the CAMs of all eight embryos tested within the first 30 s post application, as exemplified in Figure 9. To determine whether the severe toxicity observed when the essential oils were diluted in GA was, indeed, attributable to the solvent, we evaluated the membranes exposed to the pure essential oils. We found that although the pure essential oils were highly concentrated compared with the tested dilutions, they exhibited low toxicities. None of the eight CAMs evaluated showed lysis, hemorrhage, or coagulation within the first 2 min of exposure, which contrasts with the results observed in the other treatments. We provide a robust scientific foundation for the topical use of essential-oil-based sanitizers on eggshells, reaffirming that this management approach is safe and, in cases of embryonic contact, minimal and non-toxic. Exposure to GA, which is used as a diluent, did not significantly affect the embryonic viability; if excessive or prolonged penetration occurred, we would have observed elevated embryo mortality associated with massive hemorrhages, coagulation, and lysis of blood vessels. These findings underscore that sanitizing with essential oils diluted in GA is a safe practice. However, under no circumstances should GA be used as a diluent for injectable applications because of its potential risk of embryonic damage. For injecting essential oils into eggs, we strongly recommend using alternative and proven safe diluents. For example, the medium-chain triglycerides used as a diluent for essential oils in hen’s egg test chorioallantoic membrane (HET-CAM) assays did not induce irritation to the CAM, as evidenced by the absence of hemorrhage, coagulation, and vasoconstriction [47].

## 3. Materials and Methods

The CAEO (extracted from the fruit peel by pressing) (Phytoterápica^®^, Rio de Janeiro, Brazil), OBEO (extracted from the leaves and flowers by steam distillation) (BioEssência^®^, São Paulo, Brazil), and ASEO (extracted from the rhizomes by steam distillation) (Laszlo^®^, Minas Gerais, Brazil) were purchased commercially. The chemical composition of each essential oil was identified via gas chromatography coupled with mass spectrometry. The three main compounds in the CAEO were D-limonene (18.32%), p-cymene (18.04%), and limonene 1,2-diol (7.10%). In OBEO, the main constituents were estragole (69.57%), linalool (20.70%), and (E)-α-bisabolene (2.22%) (BioEssência^®^, São Paulo, Brazil). In the ASEO, the highlights were di-2-propenyl-trisulfide (30.72%), diallyl disulfide (18.95%), and methyl and allyl trisulfide (11.84%) [48].

For this antibacterial assay [49], *S. aureus* and *E*. *coli* (100 μL; optical density: 0.5 McFarland; American Type Culture Collection, Manassas, VA, USA) were inoculated separately on Mueller–Hinton agar (Difco, BD, Sparks, MD, USA). Sterile disks (6 mm in diameter; Laborclin, Pinhais, Paraná, Brazil) were supplemented with 10 μL of CAEO, OBEO, or ASEO. These disks, including the antibiotic (azithromycin at 15 μg; Laborclin, Pinhais, Paraná, Brazil) and sterilized distilled water disks, were transferred to the surface of the already inoculated Mueller–Hinton agar (Difco, BD, Sparks, MD, USA) and incubated at 36 °C for 24 h. Inhibition zones were measured to confirm the antibacterial activities. A procedure similar to that described above was also used to evaluate the minimum inhibitory concentrations (MICs) of the essential oils. In a Petri dish containing 100 µL of the bacterial suspension seeded on Muller–Hinton agar (Difco, BD, Sparks MD, USA), sterile disks soaked with 10 µL of CAEO, OBEO, or ASEO diluted in 0.5% Tween 80 at twenty concentrations ranging from 300 to 0.0003 mg/mL were fixed. Tween 80, at a concentration of 0.5%, served as a negative control, and azithromycin at 15 μg (Laborclin, Pinhais, Paraná, Brazil) served as a positive control. The plates were incubated for 24 h at 36 °C.

The three lowest effective concentrations of each essential oil, determined via the disk diffusion method, were tested in triplicate via the broth dilution method to confirm the effectiveness of these concentrations [50]. In this test, each essential oil was diluted until it reached the three desired concentrations in test tubes containing Mueller–Hinton broth (Difco, BD, Sparks, MD, USA), followed by the addition of the bacterial strains. In one of the tubes, the essential oil was not diluted, serving as a negative control. After the tubes were incubated at 36 °C for 24 h, the efficiency of each oil was visually measured in the absence of bacterial growth. The MIC value was recorded as the lowest concentration of each essential oil that inhibited bacterial growth.

**Figure 10:**
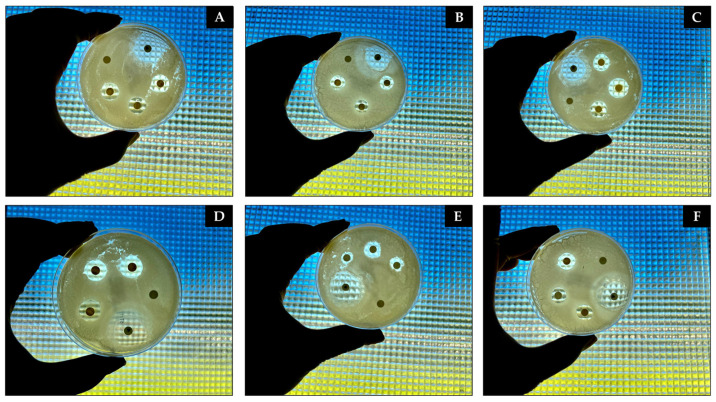
Photographic images of the disk diffusion assay results illustrating the initial antibacterial effects of CAEO, OBEO, and ASEO after 24 h of incubation at 36 °C. (**A**) Inhibition zone produced by ASEO against *E. coli*. (**B**) Inhibition zone observed for OBEO against *S. aureus*. (**C**) Inhibition zone generated by CAEO against *E. coli*. (**D**) Inhibition zone resulting from ASEO against *S. aureus*. (**E**) Inhibition zone of OBEO against *E. coli*. (**F**) Inhibition zone formed by CAEO against *S. aureus*.

The essential oils inhibited both bacteria (Figure 10), depending on the concentration tested. CAEO demonstrated inhibitory activity against *E. coli* at concentrations ranging from 300 to 9.38 mg/mL, with inhibition zones between 28.33 and 9.33 mm. Similarly, it was effective against *S. aureus* at concentrations ranging from 300 to 1.17 mg/mL, with inhibition zones ranging from 30.33 to 7.67 mm. OBEO demonstrated antimicrobial activity against *E. coli* at concentrations ranging from 300 to 1.17 mg/mL, with inhibition zones ranging from 16.67 to 7.00 mm. For *S. aureus*, the concentrations ranged from 300 to 4.69 mg/mL, with inhibition zones between 12.67 and 7.67 mm. ASEO oil exhibited a potent inhibitory effect on *E. coli* at concentrations ranging from 300 to 0.29 mg/mL, with inhibition halos ranging from 36.33 to 7.67 mm. The inhibition of *S. aureus* occurred at concentrations ranging from 300 to 1.17 mg/mL, with halos ranging from 32.00 to 8.00 mm. As a reference, the positive control showed average inhibition zones of 23.67 mm for *E. coli* and 22.00 mm for *S. aureus*. Although higher concentrations may be more effective at reducing eggshell contamination [51], they can also result in increased operating costs, potential damage to the integrity of the shell’s cuticular layer [52], and the blockage of pores [53] and a consequent reduction in the hatchability rate [12,53]. The lowest concentration of each essential oil tested, which demonstrated effectiveness in the disk diffusion method, also proved to be effective in the broth dilution method. Therefore, to prepare each essential-oil-based sanitizer, the lowest concentration of each oil that was effective against both bacteria was selected.

A flock of 58,138 breeder hens from a commercial farm located in Planaltina, the Federal District, Brazil, was subjected to rigorous and comprehensive testing. The results confirmed that the flock was completely free of *Salmonella* spp., *Mycoplasma gallisepticum*, and *Mycoplasma synoviae*, ensuring the high quality and safety of the collected eggs. This conclusion is based on extensive bacteriological and serological analyses of samples, including serum, egg, meconium, and cloacal swabs. These tests provide a reliable foundation for the subsequent experimental procedures [54,55].

A total of 1695 hatching eggs from broiler breeders (flock 27) aged 57 weeks, from the Pescoço Pelado Vermelho (PSÇ) lineage, were donated by this commercial farm. The accumulation of fecal matter and feed residue on the surface of hatching eggs can inactivate the antimicrobial properties of sanitizers or impair the interactions between them and microorganisms [27,56,57,58]. When collecting, the professionals at the donor farm discarded all the eggs that were cracked, broken, and/or had excess organic matter. Therefore, all the donated eggs were clean and intact and were subjected to sanitization using GA, FA, CAEO, OBEO, or ASEO. A control group was not subjected to any sanitization with the mentioned products. A total of 275 hatching eggs were used and appropriately identified for each treatment.

Before sanitization, the internal quality of the hatching eggs was initially tracked to assess the viability of the embryonic microenvironment and ensure that these eggs were as healthy as possible. For this analysis, 30 eggs were identified, weighed, and then carefully opened using scissors. The contents were gently dispersed on a glass surface. Initially, the height of the albumen was measured using a digital caliper. The yolk was subsequently separated from the albumen with a yolk separator, and its diameter and height were measured with a digital caliper. To complete the analysis, the pH values of the albumen and yolk of all the eggs were measured using a calibrated pH meter. The final results were obtained by applying the collected data to the formulae for the Haugh unit (HU = 100 log (albumen height + 7.57 − 1.7 egg weight^0.37^) [59] and the yolk index (YI = yolk height/yolk diameter) [60].

The eggs intended for the microbiological analysis were immediately placed in sterile bags (Labplas, Sainte-Julie, QC, Canada) after collection and stored under refrigeration (approximately 5 °C) until the analysis, which was carried out 24 h later. The microbiological quality characteristics of the contents of 15 hatching eggs were evaluated using a method similar to that described by Figueiredo et al. [61], with some modifications. For the analysis, 15 mL of the egg content was collected. These samples were placed in labeled sterile plastic bags (Labplas, Sainte-Julie, QC, Canada) to which 135 mL of 0.1% peptone water (Laborclin, Pinhais, Paraná, Brazil) was added. The plastic bags containing the solutions were then homogenized for two minutes. Serial dilutions of the homogenized solutions were carried out in 0.1% peptone water (Laborclin, Pinhais, Paraná, Brazil). The bacterial load was then quantified by plating 100 μL of the dilutions on plate count agar and red violet bile glucose agar (Ionlab, Araucária, Paraná, Brazil). These culture media were selected for counting the total aerobic mesophilic bacteria and Enterobacteriaceae, respectively. The plates were incubated at 36 °C for a period of 48 h. After this interval, the numbers of developed colonies were recorded and expressed as decimal logarithms (log_10_).

To prepare the sanitizers, each essential oil was carefully diluted in GA until it reached the following final concentrations: CAEO, 9.38 mg/mL; OBEO, 4.69 mg/mL; and ASEO, 1.17 mg/mL. The concentration of the GA used as a vehicle for the essential oils, was 93.8%. FA, at a concentration of 5 g/m^3^, was applied to the eggs through fumigation in a closed environment for 15 min, following the routine standards established at the farm. The liquid sanitizing solutions were applied to the eggs (~2.5–3 mL/egg) via hand sprayers. The eggs were left to dry naturally at room temperature for approximately 30 min. The non-sanitized eggs were kept under similar conditions. From preparing the solutions to their application, the professionals involved were properly equipped with masks, gloves, protective glasses, lab coats, and caps. According to Franco et al. [62], eggs must be sanitized within 30 min after collection, as delays in cleaning can favor a reduction in chicken viability because of contamination. All the eggs were sanitized simultaneously 20 min after collection and then sent to an experimental laboratory for artificial incubation.

**Figure 11:**
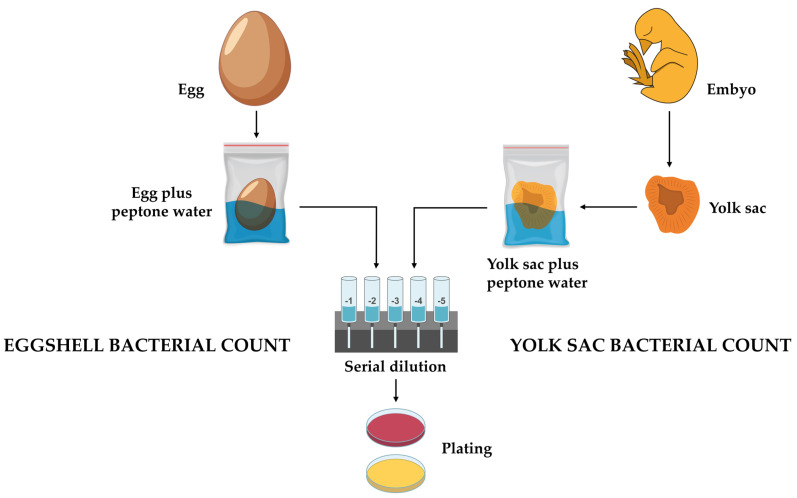
Detailed schematic of the bacterial count protocol for eggshells and yolk sacs. This diagram illustrates the process from sample extraction (egg or embryo/yolk sac) to the plating of homogenized solutions, ensuring the precise quantification of the bacterial presence. Yellow plate: mesophilic bacteria count; red plate: Enterobacteriaceae count.

We evaluated the bacterial counts on the eggshells, following a methodology similar to that used in this study for analyzing the microbiological content of the eggs, with adaptations from the protocol described by Shahein and Sedeek [36] (Figure 11). We subjected hatching eggs (nine eggs/treatment) to washing for two minutes in 0.1% peptone saline solution (Laborclin, Pinhais, Paraná, Brazil) and placed them in sterile transparent bags (Labplas, Sainte-Julie, QC, Canada). Each bag was filled with 165 mL of the solution along with three eggs/treatment. Serial dilutions of the washing solutions were carried out in 0.1% peptone water (Laborclin, Pinhais, Paraná, Brazil). We conducted this analysis in triplicate per treatment immediately after sanitization on the ninth and 18th days of incubation.

We prepared eight eggshells from each treatment for scanning electron microscopy (SEM) analysis following the protocol established by Mahato et al. [63]. After the internal contents were removed, the eggshells were carefully washed with distilled water. The eggshell membrane was then removed using tweezers. A small equatorial section of the shell, approximately 5 mm × 5 mm in size, was subsequently extracted and boiled in a 2% sodium hydroxide solution for 10 min to remove any residual membrane. The samples were rinsed again with distilled water and left to air-dry at room temperature for 12 h. The dried samples were then metalized and examined under a JEOL JSM-7001F scanning electron microscope (Jeol Ltd., Akishima, Tokyo, Japan) at standard magnifications of ×100, ×500, ×1000, and ×3000.

**Figure 12:**
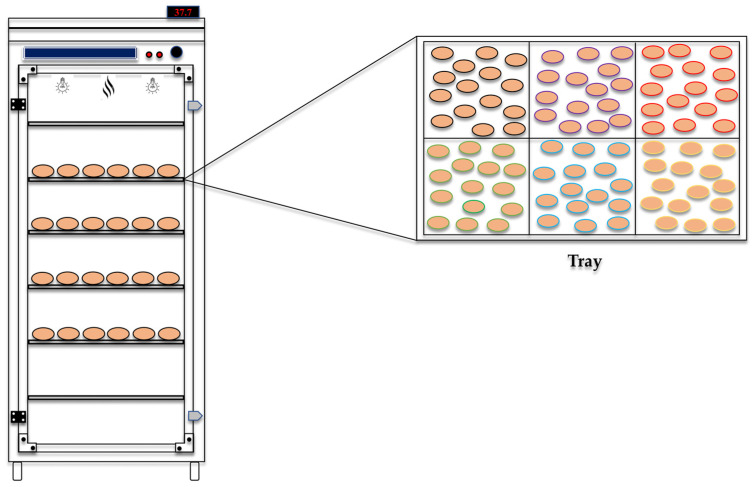
Schematic representation of egg incubation under different experimental conditions in the setter. In this figure, we present a detailed illustration of the arrangement of the hatching eggs inside the setter, highlighting each treatment’s organization and specific environment, which may vary across trays on the basis of randomness. The colored outlines clearly identify the six different treatments applied during this study, facilitating the distinction of and comparison between the experimental conditions.

Before the hatching eggs arrived at the experimental laboratory, the egg storage chamber and setters were rigorously cleaned and sanitized. The storage chamber and setters were initially washed with water and detergent. Then, the sanitizer Lysoform (2.5%; SC Johnson, Racine, WI, USA) was applied and left to act for at least 10 min, according to the manufacturer’s recommendation. The eggs were stored for 24 h in a storage chamber with a controlled temperature between 19 and 21 °C and a relative humidity between 50 and 60%. After the storage period, the incubation and hatching methodology adopted by Oliveira et al. [64] was used. Briefly, the eggs were weighed after storage and incubated in single-stage setters (Chocmaster, Curitiba, Paraná, Brazil), as illustrated in Figure 12. A total of four setters were used. In each incubation tray, the eggs from each treatment group were distributed randomly. During incubation, the mean temperature was maintained at 37.7 °C, and the mean relative humidity was 60%. Egg turning occurred automatically every hour, as recommended by Oliveira et al. [65]. Candling was performed individually on each egg on the 10th day of incubation, allowing for the removal of infertile eggs and those with embryonic mortality. During the birth period, the mean temperature was adjusted to 36.6 °C, and the mean relative humidity was adjusted to 65%. The climatic variables of the incubation room were monitored every five minutes via a HOBO data logger (Onset, Bourne, MA, USA) during the 21 days of incubation, ensuring the optimal functioning of the setters.

The parameters used to evaluate the feasibility of the egg sanitization during incubation and hatching were as follows: egg weight loss (%), hatchability of fertile eggs (%), early dead (%), mid dead (%), late dead (%), and contaminated eggs (%), according to the methodology described by Baylan et al. [66], with very few changes. To evaluate the quality of the hatched chicks, we used the Pasgar score, which considers the reflexes, navel, legs, and beak, according to the methodology described by Boerjan [67]. Next, the bodyweights of the same chicks were measured using a high-precision analytical scale (Gehaka, São Paulo, São Paulo, Brazil).

In accordance with the methodology of Upadhyaya et al. [68], the bacterial load of the yolk sac contents was determined. Seven chick embryos from each treatment were removed from the setter at 18 days of development and euthanized by cervical dislocation by a trained researcher, with the aim of collecting the contents of the yolk sac. To ensure the sterility and integrity of the procedure, the bags containing the yolk sac contents were transferred to sterile transparent bags (Labplas, Sainte-Julie, QC, Canada), each containing 150 mL of 0.1% peptone saline solution (Laborclin, Pinhais, Paraná, Brazil). The contents were then homogenized for two minutes. The homogenized solutions were subjected to serial dilutions following a careful and standardized protocol. This procedure employed the same microbiological counting methods as those used previously (Figure 11).

In this investigation, six embryos from each treatment group at 18 days of development were euthanized by cervical dislocation to assess the effects of the sanitizing solutions on the poultry respiratory system. In line with ethical and methodological standards, necropsies were conducted, and tissue samples from the tracheae and lungs were collected. These samples were fixed in 10% buffered formalin (pH 7.0), embedded in paraffin, and stained with hematoxylin and eosin (H&E). A semi-quantitative analysis of the pathological changes in all the tissues was performed, and the results were categorized as absent (−), mild (+), moderate (++), or severe (+++). Epithelial cell degeneration/necrosis, goblet cell hyperplasia, and lymphocytic inflammation were the main lesions evaluated in the tracheae (adapted from Hayretdağ and Kolankaya [46]). Bronchial epithelial degeneration/necrosis and congestion were histologically assessed in the lungs.

We measured the toxicity profiles of the sanitizing solutions on embryos via HET-CAM [69]. A total of 120 non-sanitized hatching eggs (mean weight: 56.33 ± 3.64 g) from 51-week-old PSÇ-lineage broiler breeders (batch 21) were incubated until the 9th day of embryonic development, under the same conditions described in the “incubation and hatching” section, in a single-stage setter (Premium Ecologica, Belo Horizonte, Minas Gerais, Brazil). After this period, all the eggs were subjected to candling to confirm embryonic development. In embryonated eggs, an opening was made in the shell above the air chamber with the help of surgical scissors. After being moistened with a 0.9% saline solution, the eggshell membranes were carefully removed to expose the CAMs. Then, 200 μL of each sanitizing solution, at the same concentration used to sanitize the eggshells, was pipetted onto the membrane. Each sanitizing solution was applied to eight eggs, except for the treatments with the essential oils, which were applied to 16 eggs: eight with GA as the carrier and eight without GA as the carrier. The harmful effects of the sanitizing solutions were evaluated based on the degrees of hemorrhage, coagulation, and lysis of the blood vessels of the CAMs, as described in detail in the study by Derouiche and Abdennour [70]. Observations, after the sanitizing solutions were applied, were carried out with the aid of a magnifying glass, allowing for detailed monitoring of their effects on the CAMs.

We conducted the experiment in accordance with a completely randomized design, which included six treatments and four repetitions in each treatment group, with 60 eggs per repetition. We performed the bacterial counts of the eggshells in triplicate, and the bacterial analysis of the yolk sac was conducted in seven repetitions, with each embryo considered as an independent experimental unit. Analysis of variance was performed via PROC GLM. Tukey’s test was used to compare the treatment means (*p* < 0.05). We used SAS Studio University Edition software (SAS Inst. Inc., Cary, NC, USA) for all the statistical analyses.

## 4. Conclusions

The natural and safe sanitization plan using CAEO, OBEO, and ASEO, as tested and described in this study, provides effective bacterial control for hatching eggs, increasing hatching rates, and resulting in healthier poultry without morphological alterations. This work reaffirms the promise of essential oils as a feasible solution for the sanitary management of hatching eggs. The early use of essential oils during the pre-incubation phase for the bacterial treatment of the eggshells was driven by the pursuit of safer and more effective interventions in the poultry sector. The high efficiency of the essential oils at low concentrations, as reflected in the reduced amounts needed for formulating sanitizers, can help to reduce the higher costs associated with these products compared to some synthetic alternatives, which could hinder their use. Another point to consider is that the combined application of essential oils with practical, economical, and efficient sanitization methods can eliminate the need to increase the concentrations of essential oils in egg-hatching sanitization practices, thereby minimizing the undesirable economic effects associated with the use of higher concentrations. Another important aspect for making them economically feasible is the selection of oils extracted from plants cultivated in various parts of the world or those that are easily accessible, such as CAEO, OBEO, and ASEO. Our findings expand the understanding of the topical application of essential oils to hatching eggs. According to the results of this study, we suggest a detailed protocol for sanitizing hatching eggs with essential oils (Table 7). Considering concerns regarding bacterial resistance, we recommend the cautious use of essential oils and the adoption of proper application methodologies and a management plan that promotes the periodic rotation of at least two different essential oils, along with frequent monitoring of the microbiota present on the eggshells.

## Figures and Tables

**Table 1:** Internal evaluation of eggs during pre-incubation.

Analysis	Result
Egg weight (g) *	58.83 ± 4.08
Albumen height (mm) *	6.70 ± 1.02
Haugh unit *	80.24 ± 6.34
Yolk index *	0.40 ± 0.03
Albumen pH *	8.14 ± 0.25
Yolk pH *	6.07 ± 0.15
Mesophilic bacteria (log_10_ CFU/mL) **	2.22 ± 0.38
Enterobacteriaceae (log_10_ CFU/mL) **	1.89 ± 0.15

The results are presented as the means ± standard deviations of 30 * or 15 ** eggs.

**Table 2:** Bacterial counts of eggshells during pre-incubation and incubation.

**Sanitizer**	**Mesophilic Bacteria**		
	Day 0	Day 9	Day 18
NE	3.71 ± 0.67 ^aB^	4.40 ± 0.44 ^aAB^	5.74 ± 0.28 ^aA^
GA	3.01 ± 0.72 ^abA^	3.48 ± 0.18 ^aA^	4.20 ± 1.09 ^abA^
FA	1.78 ± 0.56 ^bB^	2.90 ± 0.54 ^abAB^	4.03 ± 0.26 ^bA^
CAEO	1.12 ± 0.42 ^bA^	1.76 ± 0.18 ^bcA^	2.31 ± 0.46 ^cA^
OBEO	1.15 ± 0.53 ^bA^	1.51 ± 0.12 ^bcA^	2.10 ± 0.48 ^cA^
ASEO	1.01 ± 0.05 ^bA^	1.19 ± 0.34 ^cA^	1.98 ± 0.23 ^cA^
*p* value	<0.0001	<0.0001	<0.0001
**Sanitizer**	**Enterobacteriaceae**		
	Day 0	Day 9	Day 18
NE	1.59 ± 0.39 ^aB^	2.56 ± 0.37 ^aAB^	2.96 ± 0.21 ^aA^
GA	1.41 ± 0.37 ^aA^	1.99 ± 0.19 ^aA^	2.03 ± 0.57 ^abA^
FA	0.00 ± 0.00 * ^bC^	1.00 ± 0.45 ^bB^	2.42 ± 0.49 ^aA^
CAEO	0.00 ± 0.00 ^bA^	0.00 ± 0.00 ^cA^	0.92 ± 0.20 ^cA^
OBEO	0.00 ± 0.00 ^bB^	0.54 ± 0.47 ^bcAB^	1.08 ± 0.68 ^bcA^
ASEO	0.00 ± 0.00 ^bA^	0.00 ± 0.00 ^cA^	0.35 ± 0.60 ^cA^
*p* value	<0.0001	<0.0001	<0.0001

^A–C; a–c^ Different uppercase (row) or lowercase (column) letters indicate significant differences among means (*p* < 0.05). Non-sanitized eggs—NE; grain alcohol—GA; formaldehyde—FA; *Citrus aurantifolia* essential oil—CAEO; *Ocimum basilicum* essential oil—OBEO; *Allium sativum* essential oil—ASEO. * Indicates a count below detection limits of <10 CFU/mL. The results are presented as the means (log_10_ CFU/mL) ± standard deviations of triplicate measurements.

**Table 3:** Analysis of eggs’ weight losses, hatchability of fertile eggs, embryonic mortality, and percentage of contaminated eggs throughout the incubation cycle.

Sanitizer	Egg Weight Loss	Hatchability	Dead			
			Early	Mid	Late	Contaminated
NE	12.88 ± 1.41 ^b^	81.30 ± 2.88 ^ab^	3.90 ± 1.43 ^b^	0.86 ± 1.49 ^a^	7.03 ± 6.58 ^a^	6.91 ± 3.18 ^a^
GA	13.65 ± 1.15 ^ab^	82.56 ± 3.21 ^ab^	7.70 ± 3.21 ^ab^	1.70 ± 1.20 ^a^	6.35 ± 3.15 ^a^	1.68 ± 1.20 ^b^
FA	15.11 ± 1.46 ^a^	75.34 ± 5.87 ^b^	15.74 ± 6.70 ^a^	1.27 ± 1.41 ^a^	5.95 ± 2.54 ^a^	1.70 ± 2.08 ^b^
CAEO	13.71 ± 0.99 ^ab^	89.58 ± 2.62 ^a^	2.59 ± 1.91 ^b^	1.28 ± 1.41 ^a^	6.12 ± 3.68 ^a^	0.43 ± 0.75 ^b^
OBEO	12.91 ± 1.17 ^b^	88.31 ± 4.61 ^a^	4.33 ± 1.99 ^b^	0.86 ± 0.86 ^a^	6.50 ± 2.65 ^a^	0.00 ± 0.00 ^b^
ASEO	13.80 ± 1.18 ^ab^	87.83 ± 5.49 ^a^	3.47 ± 2.16 ^b^	2.19 ± 2.28 ^a^	6.51 ± 2.32 ^a^	0.00 ±0.00 ^b^
*p* value	0.0221	0.0058	0.0015	0.8737	0.9995	0.0006

^a,b^ Different lowercase (column) letters indicate significant differences among means (*p* < 0.05). Non-sanitized eggs—NE; grain alcohol—GA; formaldehyde—FA; *Citrus aurantifolia* essential oil—CAEO; *Ocimum basilicum* essential oil—OBEO; *Allium sativum* essential oil—ASEO. The results are presented as the means ± standard deviations of 240 eggs.

**Table 4:** Bacterial counts in the yolk sacs of the embryos at 18 days of development.

Sanitizer	Mesophilic Bacteria	Enterobacteriaceae
NE	3.21 ± 0.30 ^a^	2.65 ± 0.18 ^a^
GA	2.94 ± 0.52 ^ab^	2.14 ± 0.15 ^ab^
FA	2.93 ± 1.00 ^ab^	2.36 ± 0.29 ^a^
CAEO	1.52 ± 0.39 ^bc^	1.24 ± 0.29 ^bc^
OBEO	1.66 ± 0.47 ^bc^	1.11 ± 0.61 ^c^
ASEO	1.10 ± 0.20 ^c^	1.07 ± 0.27 ^c^
*p* value	0.0014	0.0002

^a–c^ Different lowercase (column) letters indicate significant differences among means (*p* < 0.05). Non-sanitized eggs—NE; grain alcohol—GA; formaldehyde—FA; *Citrus aurantifolia* essential oil—CAEO; *Ocimum basilicum* essential oil—OBEO; *Allium sativum* essential oil—ASEO. The results are presented as the means (log_10_ CFU/mL) ± standard deviations of seven yolk sacs.

**Table 5:** Monitoring of chick hatch weights and quality scores.

Sanitizer	Chick Weight (g) ^1^	Pasgar Score ^2^
NE	39.66 ± 0.83 ^a^	8.56 ± 1.06 ^b^
GA	38.85 ± 1.14 ^ab^	8.81 ± 0.73 ^ab^
FA	38.46 ± 0.81 ^b^	9.06 ± 0.83 ^ab^
CAEO	39.11 ± 0.65 ^ab^	9.38 ± 1.05 ^ab^
OBEO	39.42 ± 0.87 ^ab^	9.63 ± 0.70 ^a^
ASEO	39.06 ± 0.94 ^ab^	9.44 ± 0.79 ^ab^
*p* value	0.0458	0.0093

^a,b^ Different lowercase (column) letters indicate significant differences among means (*p* < 0.05). Non-sanitized eggs—NE; grain alcohol—GA; formaldehyde—FA; *Citrus aurantifolia* essential oil—CAEO; *Ocimum basilicum* essential oil—OBEO; *Allium sativum* essential oil—ASEO. ^1^ Results are the means ± standard deviations of all the chicks that hatched; ^2^ Results are the means ± standard deviations of 40 chicks.

**Table 6:** Evaluation of trachea and lung tissue samples from different treatments ^1^.

Sanitizer	Tracheal Lesion			Lung Lesion	
	Epithelial Cell Necrosis	Goblet Cell Hyperplasia	Lymphocytic Inflammation	Congestion	Bronchial Epithelial Necrosis
NE	−	−	−	−	−
GA	−	−	−	−	−
FA	−	++	+	++	−
CAEO	−	−	−	−	−
OBEO	−	−	−	−	−
ASEO	−	−	−	−	−

^1^ The data are presented in the following intensity categories: absent (−), mild (+), and moderate (++). Non-sanitized eggs—NE; grain alcohol—GA; formaldehyde—FA; *Citrus aurantifolia* essential oil—CAEO; *Ocimum basilicum* essential oil—OBEO; *Allium sativum* essential oil—ASEO. The results are the means obtained by measuring samples of six embryos.

**Table 7:** Step-by-step instructions for sanitizing hatching eggs with essential oils.

Step	Activity
1	Choose an essential oil with a proven effectiveness.
2	Add the essential oil to the correct amount of grain alcohol—the lower the concentration required for the microbial control of the eggshell the better.
3	Mix the solution carefully to ensure the proper dispersion of the essential oil.
4	After dilution, the sanitizer will be ready for use.
5	Store the sanitizer in a clean, labeled spray bottle in a cool, dark place.
6	Be sure to wear personal protective equipment and wash your hands before handling the sanitizer.
7	Apply the sanitizer as soon as possible after egg collection.
8	Choose eggs with the cleanest possible shells, avoiding those that are excessively dirty.
9	Do not wash the eggs before applying the sanitizer.
10	Position an egg so that the surface is accessible for sanitizer application.
11	Spray the sanitizer over the entire surface of the egg, ensuring uniform coverage.
12	Avoid applying excess sanitizer (use an average of 3 mL/egg).
13	Allow the eggs to dry naturally at room temperature.
14	After complete drying, store the eggs in a sanitized place until incubation.
15	When sanitization is complete, dispose of the sanitizer in a suitable manner.

## Data Availability

The raw data supporting the conclusions of this article will be made available by the authors on request.
